# Icaritin ameliorates mitochondrial dysfunction and autophagy impairment in cellular models of Alzheimer’s disease

**DOI:** 10.3389/fnagi.2026.1741339

**Published:** 2026-03-10

**Authors:** Lingqiong Xia, Tingting Liu, Zhengping Li, Xianfa Ao, Qiang Chen, Xinyu Zhou, Qianfeng Jiang, Nanqu Huang, Yong Luo

**Affiliations:** 1 Department of Neurology, The Third Affiliated Hospital of Zunyi Medical University (The First People’s Hospital of Zunyi), Zunyi, Guizhou, China; 2 Department of Pharmacy, The Third Affiliated Hospital of Zunyi Medical University (Zunyi First People's Hospital), Zunyi, Guizhou, China; 3 Longnan Hospital, The First Affiliated Hospital of Gannan Medical University (Longnan First People’s Hospital), Ganzhou, Jiangxi, China; 4 Department of Cardiovascular Medicine, The Third Affiliated Hospital of Zunyi Medical University (Zunyi First People's Hospital), Zunyi, Guizhou, China

**Keywords:** Alzheimer’s disease, autophagy dysfunction, Icaritin, mitochondrial damage, Transactive response DNA-bindingprotein 43 kDa

## Abstract

**Introduction:**

Alzheimer’s disease (AD) is the most common form of dementia, characterized by progressive memory decline, with neuropathological hallmarks including amyloid plaques and neurofibrillary tangles. Current treatments only alleviate symptoms and cannot halt disease progression. Icaritin (ICT), a natural compound, has shown neuroprotective potential. Transactive response DNA-binding protein 43 (TDP-43) is widely recognized as a key neuropathological hallmark of AD and related dementias. This study investigated the protective effects of ICT against TDP-43-induced damage in N2a/APP695swe (APP) cells and explored the underlying mechanisms.

**Methods:**

N2a/APP695swe/TARDBP cells overexpressing APP and TDP-43 were constructed via lentiviral transfection, and the optimal ICT dosage was determined using the CCK-8 assay. The effects of ICT on TDP-43 cell phenotypes were then assessed using CCK-8, ELISA, and Western blot. Finally, transmission electron microscopy, flow cytometry, assay kits, and Western blot were used to investigate the protective mechanisms of ICT.

**Results:**

ICT treatment significantly increased cell viability, reduced Aβ42 levels, and alleviated phospho-Tau and phospho-TDP-43 accumulation. Mechanistically, ICT improved mitochondrial morphology, decreased ROS levels, enhanced ATP production, and modulated the AMPK/mTOR and PINK1/Parkin autophagy signaling pathways to mitigate TDP-43-mediated cellular stress.

**Conclusion:**

ICT protects cells from TDP-43-induced mitochondrial dysfunction and autophagy impairment, providing mechanistic insight into its potential as a therapeutic agent for AD.

## 1 Introduction

Alzheimer’s disease (AD) is a prevalent neurodegenerative disorder, characterized clinically by progressive memory loss, as well as cognitive and language impairments (Jorfi et al., 2023). The neuropathological features of AD include the extracellular deposition of amyloid-β (Aβ) to form senile plaques and the intracellular aggregation of tau protein, which leads to neurofibrillary tangles. This condition typically affects individuals over the age of 60 and accounts for more than 90% of all dementia cases worldwide (Iqbal et al., 2024). In clinical practice, the primary approach to alleviate AD-related symptoms is through pharmacological interventions. Existing treatments primarily include cholinesterase inhibitors and NMDA receptor antagonists (Yiannopoulou and Papageorgiou, 2020). Cholinesterase inhibitors, such as donepezil and galantamine, are widely used in the management of AD; however, their efficacy is limited, as they only provide temporary symptom relief without altering the progression of the disease. Moreover, these drugs may cause gastrointestinal side effects and issues with tolerance, which restrict their long-term use (Yiannopoulou and Papageorgiou, 2020). NMDA receptor antagonists, such as memantine and ketamine, help to reduce neuronal damage by modulating glutamate signaling pathways, but long-term usage may lead to cognitive decline and other adverse effects (Yiannopoulou and Papageorgiou, 2020). To overcome the limitations of current therapies, researchers are actively investigating novel treatment strategies. Studies have indicated that emerging therapies, including anti-β-amyloid antibodies (Verger et al., 2023; Kang, 2024), neurotrophic factors (Tuszynski, 2024), and anti-inflammatory agents (Huang et al., 2025), may offer potential therapeutic benefits for AD. However, the goal of curing AD remains distant (Wong et al., 2019). Developing an effective treatment for AD continues to be a significant challenge in the pharmaceutical sciences.

Transactive response DNA-binding protein 43 kDa (TDP-43), encoded by the TARDBP gene, is a widely expressed protein essential for the development of the central nervous system. It is known to influence pathways involved in cell survival, metabolism, mitochondrial function, and synaptic activity (Neumann et al., 2006). Over the past decade, TDP-43 has been recognized as a critical participant in the pathogenesis of various neurodegenerative diseases (Gao et al., 2019; Xu, 2012), with significant progress made in understanding its physiological functions and role in neurodegenerative disorders (Chen-Plotkin et al., 2010). Under physiological conditions, TDP-43 is predominantly localized in the nucleus, with a small fraction shuttling between the cytoplasm and the nucleus. Under pathological conditions, such as inflammation (Correia et al., 2015), TDP-43 is released into the cytoplasm, where it regulates the formation of stress granules, ribonucleoprotein transport granules, translation, and other processes, thereby inducing neurotoxicity (Prasad et al., 2019). Released TDP-43 in the cytoplasm can undergo hyperphosphorylation and ubiquitination (Buratti, 2018), and the phosphorylated TDP-43 progressively accumulates in the form of inclusions in the cytoplasm. These insoluble inclusions in the cytoplasm suggest a disruption of the homeostatic pathways involved in mediating intracellular protein degradation (Riemenschneider et al., 2022). Neurodegenerative diseases associated with abnormal aggregation of TDP-43 are collectively referred to as ‘TDP-43 proteinopathies’ (Gao et al., 2019). Studies have shown that 57% of AD patients exhibit hippocampal p-TDP-43 inclusions, which accelerate cognitive decline and disease progression (Meneses et al., 2021); however, the specific underlying mechanisms remain incompletely understood. Although the neurotoxic mechanisms of pathological TDP-43 are not yet fully elucidated, the presence of TDP-43 in or around mitochondria, as well as its involvement in regulating mitochondrial morphology, transport, and function, suggest that mitochondria may be a key target in TDP-43 pathology (Gao et al., 2019; Wang W. et al., 2016).

Multiple studies have indicated that mitochondrial dysfunction is a major driving force in the progression of AD, accelerating the disease course by inducing neuronal dysfunction and even large-scale neuronal death (Ashleigh et al., 2023; Xie et al., 2022). In AD, various mitochondrial abnormalities have been identified, including changes in mitochondrial membrane potential, damage to mitochondrial structure, abnormalities in mitochondrial DNA (mtDNA), excessive production of reactive oxygen species (ROS), and reduced ATP synthesis, which lead to dysregulated autophagic responses in neurons and microglia (Li et al., 2022b). Moreover, mitochondrial dysfunction disrupts the normal functioning of neuronal synapses, weakening synaptic connections and reducing neurotransmission, both of which are key factors contributing to cognitive and memory impairments in AD patients (Gowda et al., 2022). In recent years, mitochondrial damage caused by the accumulation of abnormal intracellular proteins has garnered increasing attention, with one of the most widely studied proteins being TDP-43, a hallmark of neurodegenerative diseases (Yong et al., 2022). Several studies have shown that TDP-43 abnormalities can lead to mitochondrial damage, and a range of mitochondrial defects have been reported in TDP-43 proteinopathies in both cell and animal models, including damage to mitochondrial complex I (Wang W. et al., 2016; Wang et al., 2019; Liu et al., 2024), mitochondrial structural disruption (Liu et al., 2024), loss of mitochondrial membrane potential (Wang et al., 2019), reduced ATP synthesis (Wang et al., 2019), increased ROS production (Wang et al., 2019), and triggering of mtDNA release to activate neuroinflammation (Yu et al., 2020). Damaged mitochondria can activate mitochondrial autophagy, selectively isolating and eliminating impaired, aging, and dysfunctional mitochondria (Gustafsson and Dorn, 2019). Furthermore, research has shown that TDP-43 regulate with autophagy (Bose et al., 2011). Therefore, there exists a delicate balance between TDP-43, mitochondria, and autophagy: abnormal accumulation of TDP-43 can damage mitochondria and disrupt autophagic homeostasis, and mitochondrial regulation of the autophagic pathway is crucial. Mitochondrial dysfunction may disrupt the normal autophagic process, while damaged mitochondria can interfere with normal TDP-43 function, and dysfunctional autophagy cannot clear accumulated TDP-43 aggregates or damaged mitochondria (Huang C. et al., 2020). As a result, maintaining the balance between TDP-43, mitochondria, and autophagy is considered a key therapeutic target for AD treatment (Huang C. et al., 2020).

Icaritin (ICT) is an isoprenylated flavonoid compound derived from the traditional Chinese medicine Epimedium (Li et al., 2015). Several preclinical and clinical studies have demonstrated that ICT can combat various cancers, including hepatocellular carcinoma (Bailly, 2020), breast cancer (Wang et al., 2020), glioblastoma (Liu et al., 2018), and leukemia (Zhu et al., 2011). Furthermore, clinical trials of ICT have reported only a 6.9% incidence of adverse reactions, with no severe grade 3–4 events, indicating a relatively high safety profile (Fan et al., 2019). Previous studies on the mechanisms of ICT suggest that it possesses potent antioxidant and anti-inflammatory properties (Wu et al., 2014; Liu et al., 2019), and its oral administration can cross the blood–brain barrier in rats (Li et al., 2020). Our previous research has shown that ICT can alleviate AD pathology and improve disease progression. For instance, we found that ICT reduced tau pathology induced by okadaic acid (OA) in SH-SY5Y cells, improving AD (Li et al., 2022a), and it also reduced BACE1 expression in aging mice (Li et al., 2020) and APP-PS1-HEK293 cells (Feng et al., 2019), with potential mechanisms related to apoptosis pathways, such as decreasing the Bax/Bcl-2 ratio (Li et al., 2020). Additionally, ICT has been shown to mitigate mitochondrial damage and oxidative stress in TDP-43-transfected SH-SY5Y cells (Zhou et al., 2021) and inhibit autophagy (Zhou et al., 2022). However, the specific mechanisms of TDP-43 pathology in AD remain unclear, and whether ICT can alleviate TDP-43 pathology in the context of AD is yet to be determined. Therefore, we aim to evaluate whether TDP-43 transfection in APP cells exacerbates AD-related pathology and to further investigate whether the mechanisms are associated with mitochondrial damage and autophagic dysfunction, as well as whether ICT can protect against such damage.

Collectively, the evidence summarized above indicates that Alzheimer’s disease is not only characterized by Aβ and tau pathology, but is also closely associated with TDP-43 proteinopathy, mitochondrial dysfunction, and impaired autophagic homeostasis. Pathological accumulation of TDP-43 can disrupt mitochondrial structure and function, leading to excessive ROS production, ATP depletion, and synaptic dysfunction, while mitochondrial damage in turn activates dysregulated autophagy that fails to efficiently clear damaged mitochondria and TDP-43 aggregates. This reciprocal and self-amplifying interplay among TDP-43, mitochondrial dysfunction, and autophagy impairment contributes to neuronal vulnerability and disease progression in AD. Given the antioxidant, mitochondria-protective, and autophagy-modulating properties of ICT, together with its ability to cross the blood–brain barrier and its favorable safety profile, we hypothesized that ICT may exert neuroprotective effects in AD by restoring mitochondrial homeostasis and autophagic balance in the context of TDP-43–associated pathology. Based on this rationale, the present study investigates the effects of TDP-43 overexpression in APP cells and evaluates whether ICT can ameliorate AD-related mitochondrial damage and autophagy dysfunction.

## 2 Materials and methods

### 2.1 Construction of stable cell lines

#### 2.1.1 Construction of APP stable cell line

The Neuro-2a (N2a) cell line was purchased from Henan Wenteer Biotechnology Co., Ltd., which also constructed the N2a/pLVX (PLVX) and APP stable cell lines. The N2a cell clones were originally established by R. J. Klebe and F. H. Ruddle through spontaneous tumors in A-strain mice. In brief, N2a cells were infected with lentiviral vectors, including control virus (empty plasmid) and target virus (APP695swe recombinant plasmid). After 72 h of infection, puromycin was added to selectively kill cells that were not effectively infected, resulting in the selection of stable clones expressing the target gene under puromycin selection pressure. Stable clones were then confirmed by RT-qPCR.

#### 2.1.2 Construction of TDP-43 stable cell line

First, lentivirus packaging was performed using HEK-293 cells. Briefly, HEK-293 cells were seeded in a 10 cm culture dish and incubated for 16 h. The cells were then transfected using a three-plasmid system (TARDBP plasmid: pxp2X plasmid: pmd2G plasmid = 5:2.5:5) for 6 h. After transfection, the medium was replaced with DMEM high-glucose complete medium. Supernatants were collected at 48 and 72 h, combined, and mixed with 1 mL of EZ lentiviral concentrate. The mixture was incubated at 4 °C overnight, followed by centrifugation to remove the supernatant and resuspension in PBS, yielding the packaged lentivirus. The target virus was mixed with transfection reagents in the appropriate ratio, then used to infect APP cells. After 9 h, the medium was replaced with DMEM high-glucose complete medium. The transfection efficiency was observed using a fluorescence microscope (Sartorius Instrument System Co., Ltd., Beijing, China) 48 h post-infection. In this study, the transfection efficiency of the TARDBP construct was assessed using fluorescence imaging combined with quantitative analysis. Specifically, after transducing cells with a lentiviral vector carrying a GFP tag, the proportion of GFP-positive cells relative to the total cell number was calculated using fluorescence microscopy and ImageJ software.

### 2.2 Cell culture

N2a, PLVX, APP, and TDP-43 stable cell lines were cultured in high-glucose DMEM medium (Gibco, Thermo Fisher Scientific, USA; Cat. No. C11995500BT) supplemented with 10% FBS (Gibco, Thermo Fisher Scientific, USA; Cat. No. 16000044), 1% penicillin–streptomycin (Solarbio, Beijing, China; Cat. No. P1400), and incubated at 37 °C in a 5% CO₂ incubator. Cells were dissociated using 0.25% trypsin containing EDTA (Sparkjade, Nanjing, China; Cat. No. CN0004-100ML), and were subcultured or plated for subsequent assays.

### 2.3 ICT treatment of cells

TDP-43 cells were seeded in appropriate culture plates and allowed to attach overnight. To evaluate the potential therapeutic effects of ICT, cells were treated with varying concentrations of ICT (0.01, 0.1, and 1 μM) (Solarbio, Beijing, China; Cat. No. II0040) for 24 h. ICT was dissolved in DMSO (Sigma-Aldrich, USA; Cat. No. D2650), and the final concentration of DMSO in the culture medium did not exceed 0.1% in any treatment group. After the treatment period, cells were harvested for subsequent analyses, including assessments of mitochondrial function and protein expression et al. Control cells received an equivalent volume of vehicle DMSO without ICT.

### 2.4 CCK-8 assay for cell viability

Cell viability was measured using the CCK-8 kit (Solarbio, Beijing, China; Cat. No. CA1210-100). This assay is based on the WST-8 method, where, in the presence of an electron coupling reagent, it is reduced by mitochondrial dehydrogenases to form an orange-yellow water-soluble formazan compound. CCK-8 reagent was added at a 10:1 ratio, and cells were incubated for 3 h in a humidified environment at 37 °C and 5% CO₂. After incubation, cell viability was assessed using a fluorescence microplate reader (Bio-Rad Laboratories, Hercules, CA, USA) at an excitation/emission wavelength of 450 nm.

### 2.5 Western blot

Adherent cells were harvested and mixed with RIPA lysis buffer (Solarbio, Beijing, China; Cat. No. R0010) containing 1 × protein phosphatase inhibitor (Solarbio, Beijing, China; Cat. No. P1260) and PMSF (Solarbio, Beijing, China; Cat. No. P0100) to obtain cell lysates. After sonication, the supernatant was collected, and protein concentration was quantified using the BCA method (Solarbio, Beijing, China; Cat. No. PC0020). The lysates were then mixed with PBS and 5× protein loading buffer (Omni-Easy™; EpiZyme, Shanghai, China; Cat. No. LT101S) and subjected to protein denaturation (100 °C, 10 min). The samples, containing 30 μg of protein, were separated on SDS-PAGE gels (6–15%, 10-well, EpiZyme, Shanghai, China; Cat. No. LK208-211). The proteins were transferred onto a PVDF membrane (0.45 μm) (Millipore Immobilon-P PVDF membrane, MilliporeSigma, USA; Cat. No. IPVH00010) using a transfer device (Bio-Rad Trans-Blot Cell 36S Western Blot Transfer System; Bio-Rad Laboratories, Hercules, CA, USA). The membrane were blocked for 30 min with 1× non-protein blocking solution (EpiZyme, Shanghai, China; Cat. No. PS108) and incubated overnight at 4 °C with primary antibodies as follows: Anti-APP (Immunoway Biotechnology, Plano, TX, USA; Cat. No. YT5754; 1:1000), Anti-TDP-43 (ZenBio, Chengdu, China; Cat. No. Q13148; 1:1000), Anti-pTDP-43 (Ser409/410) (Proteintech, Wuhan, China; Cat. No. 80007-1-RR; 1:1000), Anti-Tau (Proteintech, Wuhan, China; Cat. No. 10274-1-AP; 1:1000), Anti-pTau (Thr217) (Proteintech, Wuhan, China; Cat. No. 31138-1-AP; 1:1000), Anti-PGC-1α (ZenBio, Chengdu, China; Cat. No. 381615; 1:1000), Anti-PINK1 (Santa Cruz Biotechnology, Dallas, TX, USA; Cat. No. sc-517353; 1:1000), Anti-Parkin (ZenBio, Chengdu, China; Cat. No. 381626; 1:1000), Anti-AMPK (Cell Signaling Technology, Danvers, MA, USA; Cat. No. 2532S; 1:1000), Anti-p-AMPK (Thr172) (Abcam, Cambridge, UK; Cat. No. ab131499; 1:1000), Anti-mTOR (Abcam, Cambridge, UK; Cat. No. ab245370; 1:1000), Anti-p-mTOR (Ser2448) (Abcam, Cambridge, UK; Cat. No. ab109268; 1:1000), and Anti-GAPDH (ZenBio, Chengdu, China; Cat. No. 511203; 1:10000). The membrane was then incubated with secondary antibody at room temperature for 1 h. Immunoreactivity was developed using Immobilon™ ECL Ultra Western HRP Substrate (EpiZyme, Shanghai, China; Cat. No. SQ201) and visualized using the Chemidoc MP Imaging System (Bio-Rad Laboratories, Hercules, CA, USA). Specific bands were quantified using ImageLab software (Bio-Rad Laboratories, Hercules, CA, USA).

### 2.6 Fluorescent quantitative reverse transcription PCR (RT-qPCR)

RNA was extracted from cells using TRIZOL (Takara, Shiga, Japan; Cat. No. 108–95-2) reagent according to the manufacturer’s instructions. RNA concentration was measured using a NanoDrop ND-1000 (Thermo Fisher Scientific, USA; NanoDrop ND-1000). Two micrograms of RNA were reverse-transcribed into cDNA using a reverse transcription kit (Takara, Shiga, Japan; Cat. No. RR037A). qPCR was performed using SYBR® Green PCR Master Mix (iScience, Beijing, China; Cat. No. EG20117T) on a CFX96 instrument (Bio-Rad Laboratories, Hercules, CA, USA). The RT-qPCR program consisted of the following steps: initial denaturation at 95 °C for 10 min; amplification by cycling at 95 °C for 10 s and 60 °C for 30 s (for 45 cycles); and a melt curve with denaturation at 95 °C for 10 s and 65 °C for 30 s.

### 2.7 Enzyme-linked immunosorbent assay (ELISA)

Aβ42 levels in cell supernatants from each group were quantified using an Aβ42 ELISA Kit (Jianglai Bio, China; Cat. No. JL11386) according to the manufacturer’s instructions. Briefly, 10 μL of anti-Aβ42 antibody was added to the samples, followed by 50 μL of streptavidin-HRP. The plates were incubated at 37 °C for 60 min. After incubation, the plates were washed 5 times with wash buffer. Then, 50 μL of substrate solution A and 50 μL of substrate solution B were added, and the plates were incubated at 37 °C in the dark for 10 min. Finally, 50 μL of stop solution was added, and the optical density (OD) at 450 nm was measured using a microplate reader.

### 2.8 Transmission Electron microscopy (TEM)

TEM was used to assess mitochondrial morphological characteristics. Briefly, cells were collected, fixed (0.1 M phosphate buffer, 2.5% glutaraldehyde, 2.5% PFA), and incubated at 4 °C for 2 h. After fixation, cells were post-fixed in 1% OsO4 for 2 h and then contrasted with 0.5% uranyl acetate for 30 min. The samples were processed according to standard procedures using Embed 812 epoxy resin (Electron Microscopy Sciences, Hatfield, PA, USA). Semi-thin cross-sectional slices (1 μm thick) were stained with Richardson’s stain and imaged using a BA210 Digital biological microscope (Motic China Group Co., Ltd.). All observations were carried out on a JEM-1400FLASH transmission electron microscope (JEOL, Japan).

### 2.9 Flow cytometry analysis

Cellular ROS levels were measured using the Abbkine ROS Detection Kit (Abbkine, Wuhan, China; Cat. No. KTB1910) according to the manufacturer’s instructions. Briefly, adherent cells were harvested and centrifuged at 1000 × g for 5 min, then resuspended in complete culture medium containing the ROS probe. Cells were incubated at 37 °C for 30 min, washed with PBS, and resuspended in 100 μL PBS. Samples were analyzed using a FACS Canto II flow cytometer (BD Biosciences, USA). FSC vs. SSC gating was applied to select the main cell population and doublets were excluded using FSC-H vs. FSC-A. ROS-positive cells were defined relative to unstained controls.

### 2.10 ATP detection

We used an ATP detection kit (Elabscience, Wuhan, China; Cat. No. E-BC-F002) to measure cellular ATP content. Briefly, cells were collected, and reagents were added at a specific ratio. After boiling in a water bath for 10 min and cooling under running water, the supernatant was collected by centrifugation. Enzyme working solution was then added, and the samples were transferred to a 96-well plate for detection.

### 2.11 Molecular docking

Briefly, the small molecule structure was drawn using ChemBioDraw Ultra 14.0, and the structure was imported into ChemBio3D Ultra 14.0 for energy minimization, with the Minimum RMS Gradient set to 0.001. The small molecule was then saved in mol2 format. The optimized small molecule was imported into AutodockTools-1.5.6 for hydrogenation, charge calculation, charge assignment, and rotatable bond setting, then saved in pdbqt format. The protein structure was downloaded from the Uniprot database, and Pymol 2.3.0 was used to remove crystallization water and original ligands. The protein structure was imported into AutoDockTools (v 1.5.6) for hydrogenation, charge calculation, charge assignment, and atom type specification, then saved in pdbqt format. Protein binding sites were predicted using POCASA 1.1, and docking was performed using AutoDock Vina 1.1.2. The parameters related to TDP43 were set as follows: center_x = −2.5, center_y = 7.0, center_z = −14.0; search space: size_x: 60, size_y: 60, size_z: 60 (with a grid spacing of 0.375 Å), exhaustiveness: 10, and the remaining parameters were set to default. Finally, PyMOL 2.3.0 was used for interaction mode analysis of the docking results.

### 2.12 Statistical analysis

All statistical analyses were performed using SPSS 29.0 (IBM SPSS Statistics Professional Software) for all experimental data. The statistical method used was one-way analysis of variance (ANOVA) for comparisons among multiple groups. Results are presented as means ± standard deviation. If variances were homogeneous, one-way ANOVA was applied for multiple group comparisons. If variances were heterogeneous, Dunnett’s T3 test was used. A significance level of *α* = 0.05 was set, and a *p*-value <0.05 was considered statistically significant.

## 3 Results

### 3.1 Successful construction of APP and TDP-43 cell models

**Figure 1:**
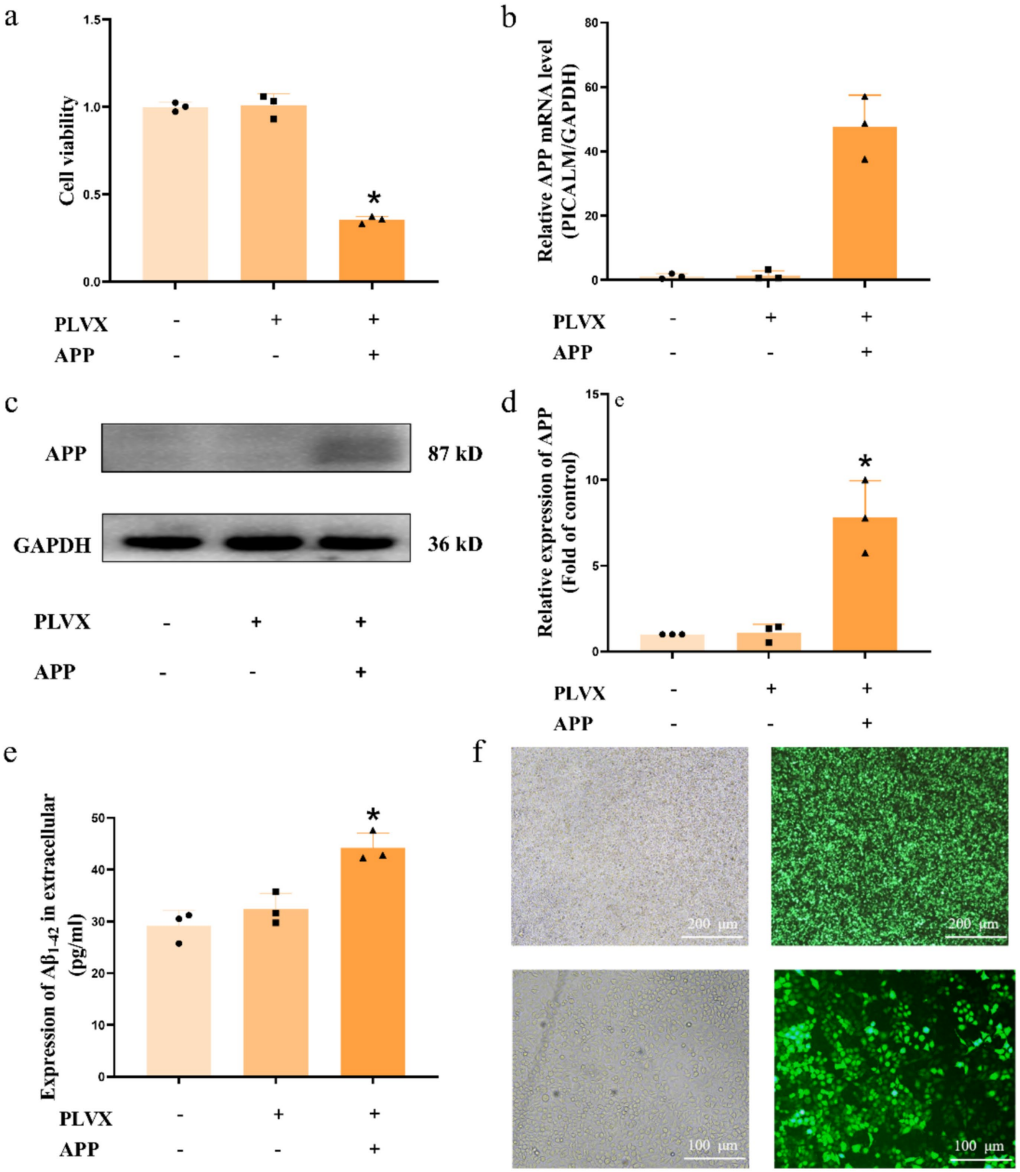
Establishment of APP and TDP-43 cell models. *X*-axis symbols ‘+’ and ‘−’ indicate the presence or absence of specific treatments or transfections, respectively. Bars with all ‘−’ symbols represent the control group (untreated or untransfected N2a cells). Subsequent symbols indicate the corresponding experimental groups as described in the figure. **(a)** Cell viability assay of N2a, PLVX, and APP cells. **(b)** RT-qPCR analysis of APP695swe gene expression in stably transfected N2a cells obtained by lentiviral infection with APP695swe or empty vector. **(c)** Western blot analysis of APP protein expression levels in N2a, PLVX, and APP cells. **(d)** Quantification of APP protein expression shown in panel **(c)**. **(e)** ELISA detection of secreted Aβ42 levels in the supernatants of N2a, PLVX, and APP cells. **(f)** Lentiviral transfection efficiency of *TARDBP* and establishment of TDP-43 stable cell line. *p < 0.05, *n* = 3, data represent three independent experiments.

We constructed an *in vitro* AD model using N2a cells infected with lentivirus carrying the APP695swe gene, and then further established an in vitro AD model with TDP-43 pathology by infecting these cells with lentivirus carrying the TDP-43 gene. First, CCK-8 assays revealed a significant decrease in cell viability following overexpression of APP695swe (Figure 1a). To verify the successful construction of the APP cell model, we performed RT-qPCR to assess the expression of the APP695swe gene, which showed a significant increase in mRNA levels (Figure 1b). Western blot analysis further confirmed that the accumulation of APP695swe mRNA led to a marked increase in APP protein expression (Figure 1c), and the quantification of the protein levels is shown in Figure 1d. ELISA analysis of the culture supernatant showed that Aβ42 levels were significantly elevated in APP cells (Figure 1e). These results confirm the successful construction of the APP cell model. To further validate the successful creation of the TDP-43 model, we observed transfection efficiency of HEK-293 cells to be >90%, indicating successful lentivirus packaging (Figure 1f). The successfully packaged lentivirus was then used to infect APP cells, with a transfection efficiency >70% (Figure 1f). These results collectively demonstrate the successful establishment of the TDP-43 cell model.

### 3.2 ICT alleviates the deterioration of AD pathology induced by TDP-43

**Figure 2:**
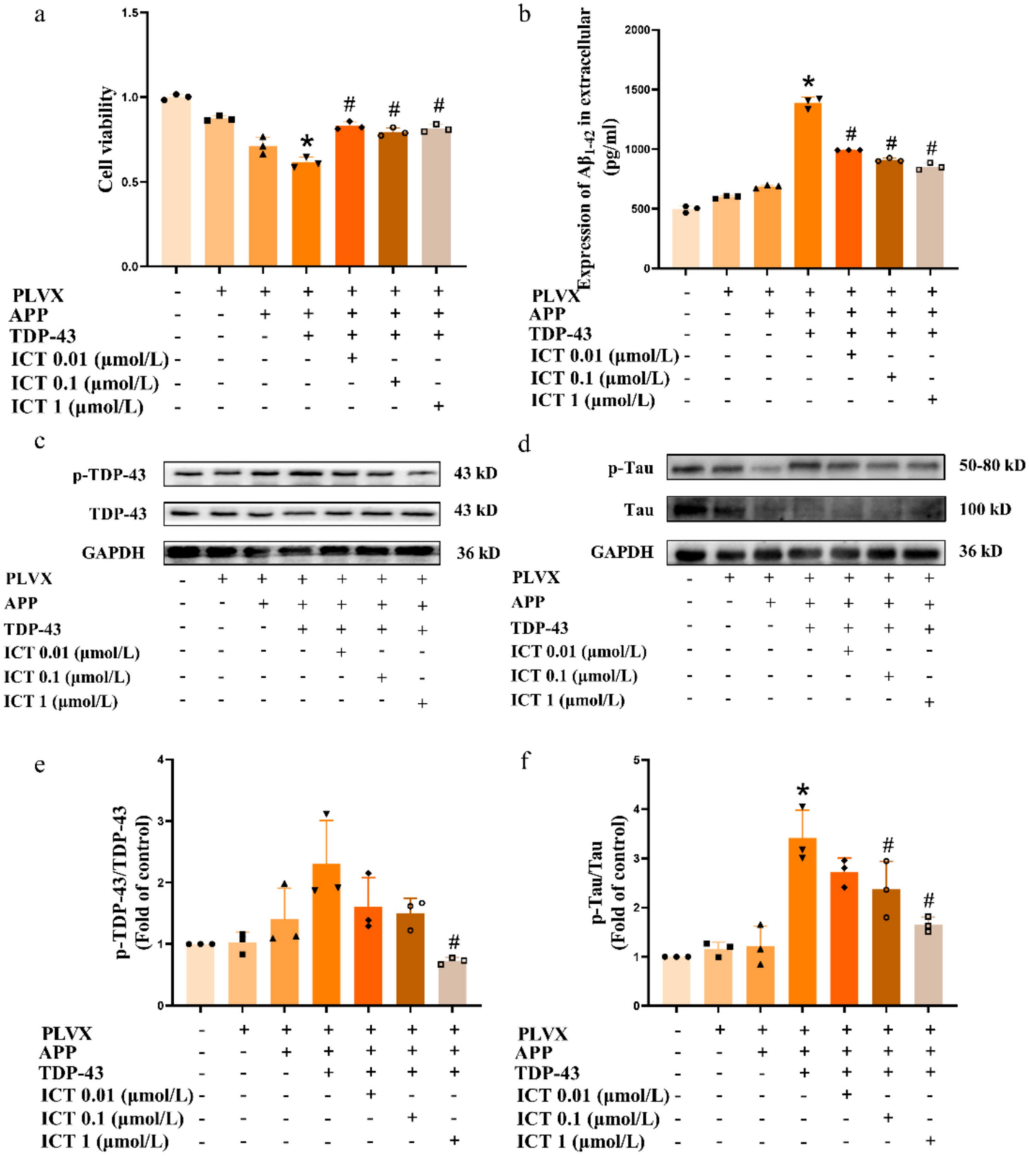
ICT attenuates the exacerbation of AD pathology induced by TDP-43 pathology. Symbols on the *X*-axis are as described in Figure 1. **(a)** Cell viability assessed by CCK-8 assay. **(b)** ELISA analysis of secreted Aβ42 levels in the cell supernatant. **(c)** Western blot analysis of the phosphorylated TDP-43 to total TDP-43 (p-TDP-43/TDP-43) ratio. **(d)** Western blot analysis of the phosphorylated Tau to total Tau (p-Tau/Tau) ratio. **(e)** Quantification of protein levels shown in panel **(c)**. **(f)** Quantification of protein levels shown in panel **(d)**. *p < 0.05, TDP-43 group vs. APP group; ^#^*p* < 0.05, ICT treatment group vs. TDP-43 group; *n* = 3, data represent three independent experiments.

To further evaluate the phenotypic changes in TDP-43 cells and investigate the effects of ICT treatment on the cell phenotype, we screened the drug concentrations for ICT treatment. After 24 h of ICT treatment on TDP-43 cells, cell viability was assessed using the CCK-8 assay. The results showed that ICT at concentrations of 0.01, 0.1, and 1 μmol/L had no cytotoxic effects, and concentrations of 5 and 10 μmol/L did not significantly affect cell viability. Therefore, 0.01, 0.1, and 1 μmol/L were selected for subsequent experiments (Supplementary Figure S1). To assess changes in cell viability, we performed the CCK-8 assay, and the results showed that overexpression of TARDBP gene significantly reduced cell viability, while ICT treatment enhanced cell viability (Figure 2a).

Amyloid Precursor Protein (APP) is cleaved by secretases to form Aβ42. Under pathological conditions, the imbalance between Aβ42 production and clearance leads to the formation of insoluble Aβ42 oligomers, which are considered the main form of neurotoxicity in AD (Sandberg et al., 2022). Previous studies have reported that TDP-43 levels are correlated with Aβ oligomer levels (Caccamo et al., 2010), and Aβ42 is the main form of Aβ oligomers (Kim et al., 2022). Thus, we measured the Aβ42 levels in the cell supernatants, and the results showed that the Aβ42 levels were significantly elevated in the TDP-43 cells compared to the APP cells (Figure 2b), and ICT treatment led to varying degrees of reduction in Aβ42 levels.

Under pathological conditions, TDP-43 can be phosphorylated (Buratti, 2018), and its phosphorylated form accumulates in the neuronal cytoplasm, leading to neurotoxicity (Yang et al., 2010). Studies have shown that overexpression of the wild-type TARDBP gene in SH-SY5Y cells is sufficient to induce the formation of p-TDP-43 pathology (Ko et al., 2024). Therefore, we assessed p-TDP-43 pathology and found that overexpression of APP695swe did not result in significant p-TDP-43 pathology. However, in the TDP-43 cells, obvious p-TDP-43 pathology was formed, which was reduced after ICT treatment (Figure 2c).

Tau pathology, one of the major pathologies in AD, is thought to synergize with TDP-43 pathology (Latimer and Liachko, 2021). Comparative analysis of brain homogenates from AD patients with and without TDP-43 pathology showed that TDP-43 pathology could increase p-Tau pathology in the brain (Tomé et al., 2023). Therefore, we further assessed p-Tau pathology in each group of cells. The results showed that overexpression of APP695swe did not form significant p-Tau pathology, whereas TDP-43 cells exhibited significant p-Tau pathology, which was reduced following ICT treatment (Figure 2d). Figure 2e shows the semi-quantitative analysis of Figure 2c, and Figure 2f shows the semi-quantitative analysis of Figure 2d.

### 3.3 ICT alleviates mitochondrial damage induced by TDP-43 pathology

**Figure 3:**
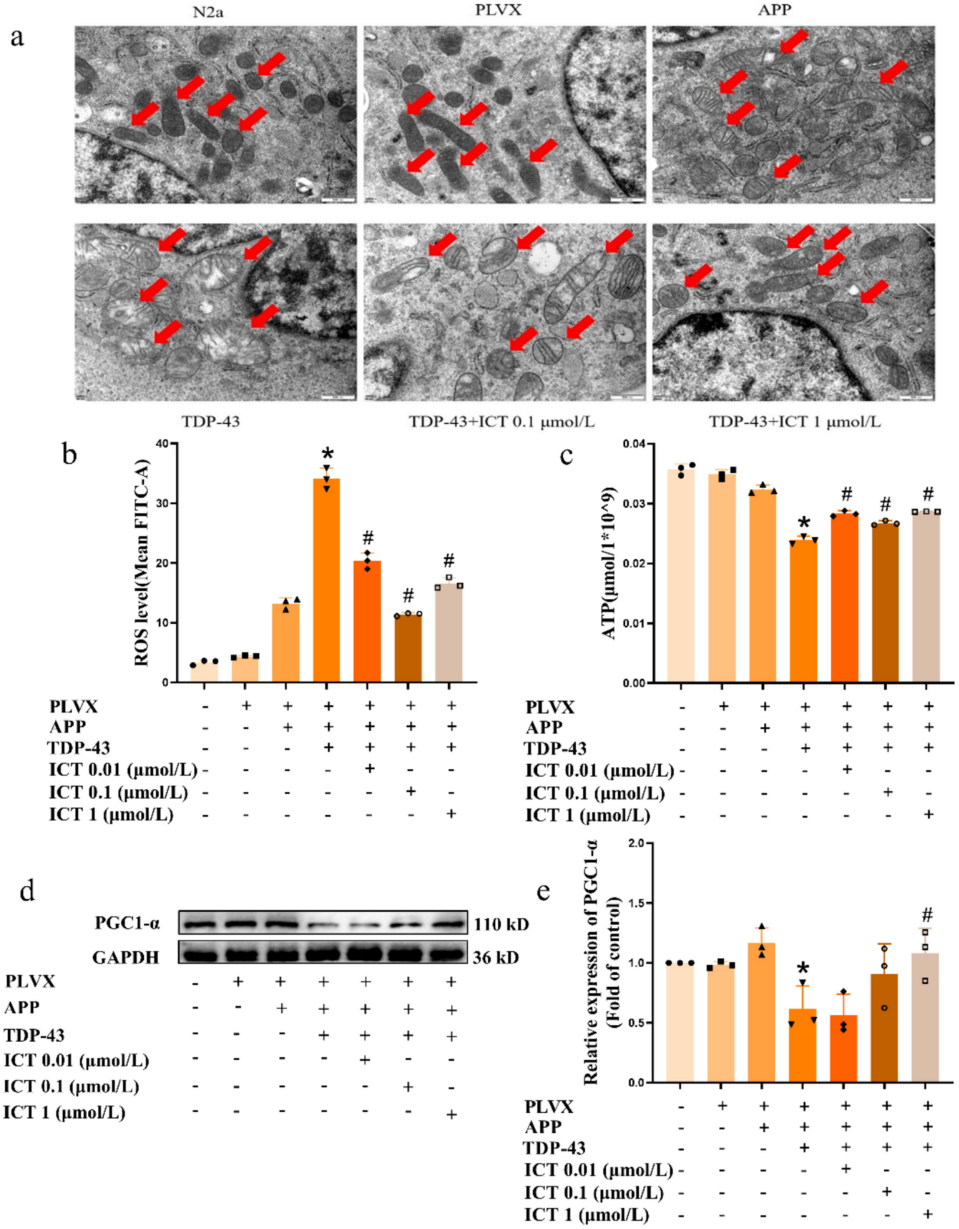
ICT alleviates mitochondrial damage induced by TDP-43. Symbols on the *X*-axis are as described in Figure 1. **(a)** Representative TEM images showing mitochondrial ultrastructural morphology. **(b)** Flow cytometric analysis of intracellular ROS levels in different groups. **(c)** Quantification of cellular ATP levels using a commercial assay kit. **(d)** Western blot analysis of PGC-1α protein expression. **(e)** Quantification of PGC-1α expression shown in panel **(d)**. *p < 0.05, TDP-43 group vs. APP group; ^#^*p* < 0.05, ICT treatment group vs. TDP-43 group; *n* = 3, data represent three independent experiments.

Previous studies have shown that mitochondria are a target of TDP-43 pathology (Wang et al., 2019; Zhou et al., 2021). To explore the impact of TDP-43 pathology on mitochondria, we used TEM to observe mitochondrial ultrastructure, flow cytometry to detect ROS expression, and ATP assay kits to assess ATP levels. TEM results revealed that after overexpression of APP695swe, mitochondria exhibited mild swelling and a slight reduction in mitochondrial cristae. However, after overexpression of APP695swe/TARDBP, mitochondria showed obvious swelling with almost complete disappearance of cristae, and this effect was reversed upon ICT treatment (Figure 3a).

Mitochondria are the primary source of ROS production (Dan Dunn et al., 2015), and defective mitochondria can lead to the accumulation of ROS (Murphy, 2009). Moreover, excessive ROS can impair neuronal survival and function (Blesa et al., 2015; Ott et al., 2007). Therefore, we examined whether TDP-43 pathology affects ROS generation in APP cells overexpressing TARDBP and quantified intracellular ROS expression using flow cytometry. The results showed that overexpression of APP695swe led to enhanced ROS expression compared to normal cells. However, overexpression of APP695swe/TARDBP significantly increased ROS expression compared to the APP695swe group, and this effect was partially reversed by ICT treatment (Figure 3b).

Additionally, ATP is primarily synthesized by the electron transport chain on the inner mitochondrial membrane (Saraste, 1999), and defective mitochondria can lead to reduced ATP production (Kaur et al., 2023). We further quantified ATP production in the cells, and the results showed no significant reduction in ATP levels after APP695swe overexpression. However, after overexpression of APP695swe/TARDBP, ATP production was significantly reduced, and ICT treatment resulted in varying degrees of increased ATP production (Figure 3c).

PGC1-α plays a crucial role in regulating mitochondrial biogenesis and oxidative stress responses. It reduces ROS-induced damage within cells, thereby protecting mitochondrial function. Therefore, we further assessed the expression of PGC1-α, a key protein involved in mitochondrial biogenesis. The results showed that after overexpression of APP695swe/TARDBP, PGC1-α expression was significantly decreased, and ICT treatment resulted in a recovery of its expression levels (Figures 3d,e).

### 3.4 ICT improves autophagy dysregulation induced by TDP-43 pathology

**Figure 4:**
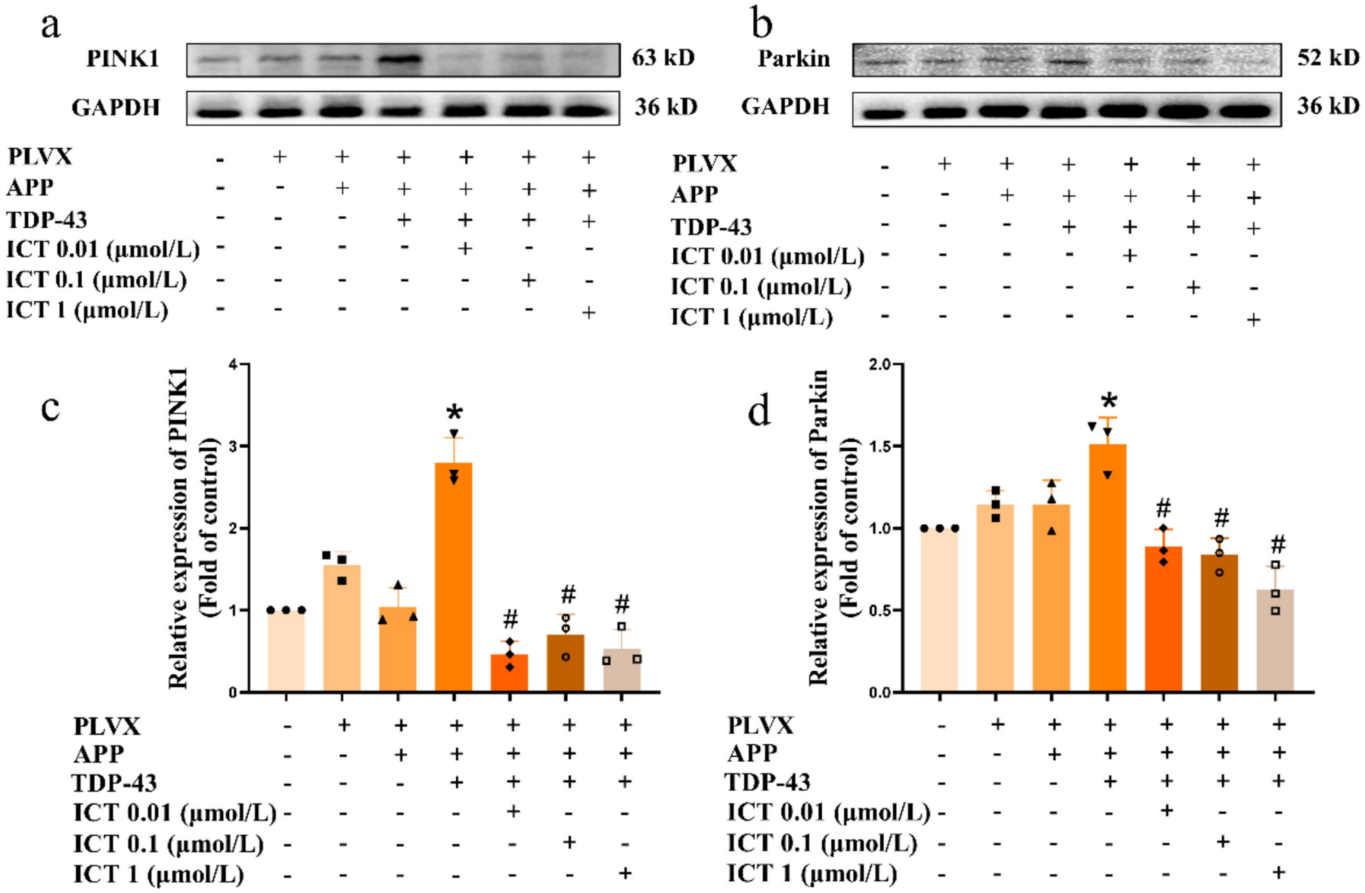
ICT attenuates the upregulation of PINK1/Parkin signaling pathway proteins induced by TDP-43 pathology. Symbols on the *X*-axis are as described in Figure 1. **(a)** Western blot analysis of PINK1 protein expression levels. **(b)** Western blot analysis of Parkin protein expression levels. **(c)** Quantification of PINK1 expression shown in panel **(a)**. **(d)** Quantification of Parkin expression shown in panel **(b)**. *p < 0.05, TDP-43 group vs. APP group; ^#^*p* < 0.05, ICT treatment group vs. TDP-43 group; *n* = 3, data represent three independent experiments.

Autophagy is a cellular phenomenon that occurs when cells experience pathological damage. Studies have shown that TDP-43 pathology co-localizes with autophagy-related proteins such as LC3 and p62/SQSTM1 (Mizuno et al., 2006; Hiji et al., 2008), suggesting a direct relationship between autophagy and TDP-43 pathology. Given the exacerbation of mitochondrial damage upon overexpression of APP695swe/TARDBP, we aimed to investigate whether mitochondrial autophagy levels were altered. As the mitochondrial autophagy signaling pathway is complex and multifaceted, the specific signaling pathways associated with TDP-43 pathology are not yet fully understood. Therefore, we focused on investigating the ubiquitin-mediated mitochondrial autophagy pathway, namely the (PTEN-induced putative kinase 1, PINK1)/(Parkin RBR E3 ubiquitin protein ligase, Parkin) pathway (Ning et al., 2022), which is considered one of the most well-studied mitochondrial autophagy pathways (He et al., 2019). The results showed that overexpression of APP695swe alone did not significantly affect the PINK1/Parkin-mediated mitophagy pathway. In contrast, co-overexpression of APP695swe and TARDBP markedly increased the expression of PINK1 and Parkin, indicating activation of the PINK1/Parkin-dependent autophagy pathway. After ICT treatment, protein expression levels were restored to normal levels (Figures 4a–d).

**Figure 5:**
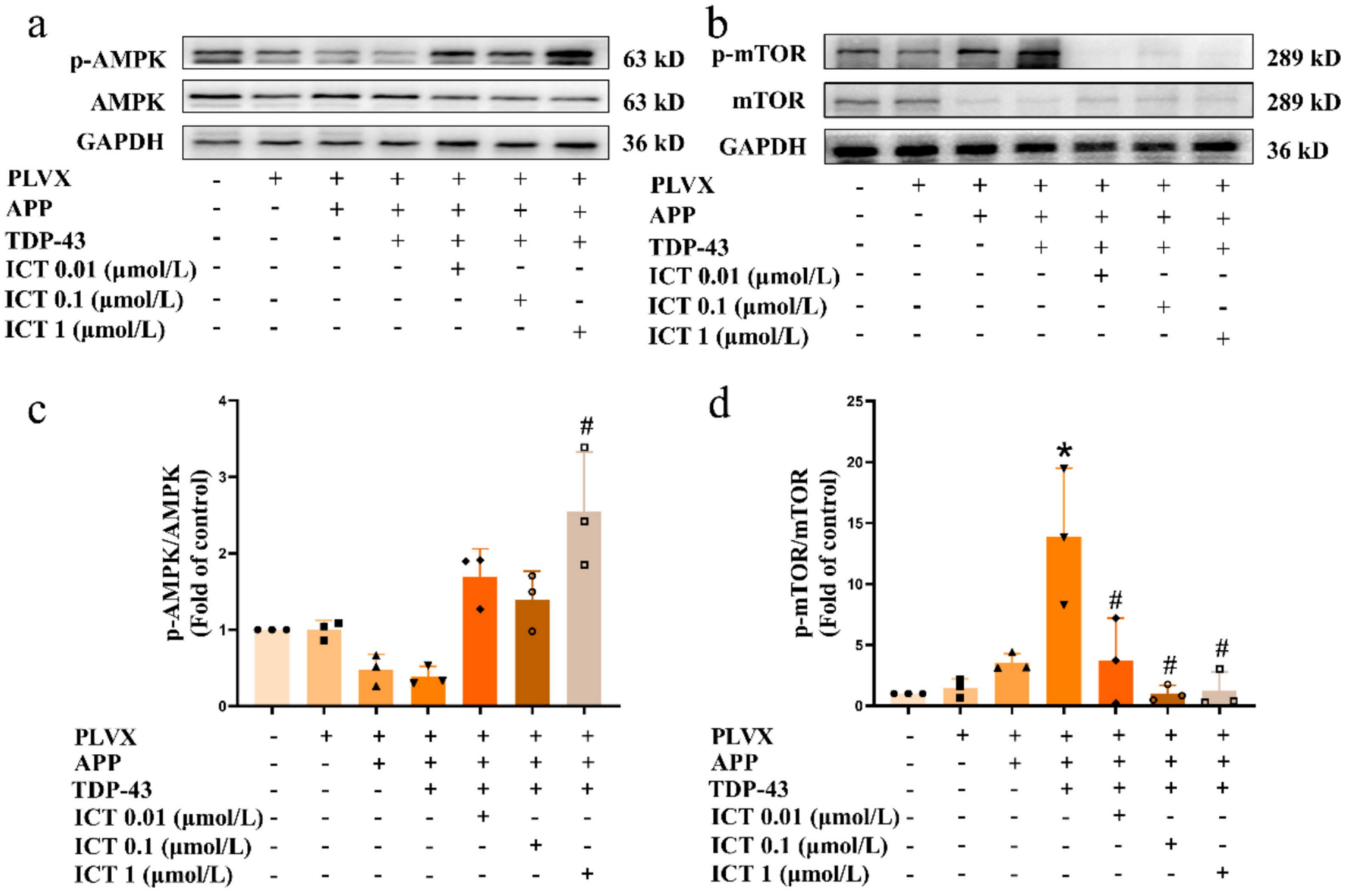
ICT alleviates the dysregulation of AMPK/mTOR signaling pathway proteins induced by TDP-43 pathology. Symbols on the *X*-axis are as described in Figure 1. **(a)** Western blot analysis of the phosphorylated to total AMPK (p-AMPK/AMPK) ratio. **(b)** Western blot analysis of the phosphorylated to total mTOR (p-mTOR/mTOR) ratio. **(c)** Quantification of protein levels shown in panel **(a)**. **(d)** Quantification of protein levels shown in panel **(b)**.*p < 0.05, TDP-43 group vs. APP group; ^#^*p* < 0.05, ICT treatment group vs. TDP-43 group; *n* = 3, data represent three independent experiments.

**Figure 6:**
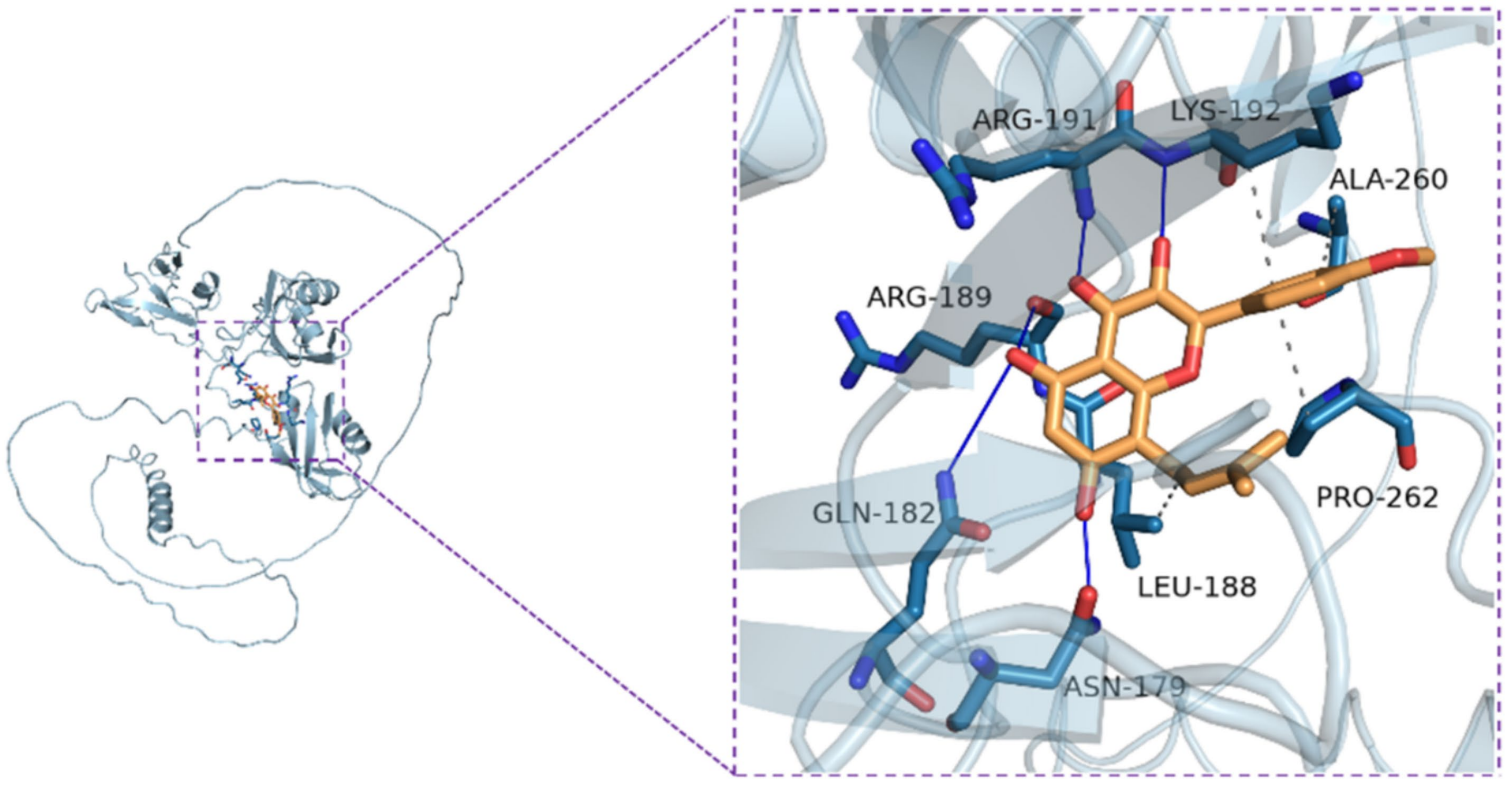
Molecular docking model of ICT with TDP-43 protein.

In light of the changes observed in the mitochondrial autophagy pathway, we further examined the activation of proteins related to the (AMP-activated protein kinase, AMPK)/(mechanistic target of rapamycin, mTOR) signaling pathway, as the AMPK/mTOR pathway is considered a central component in the autophagy regulatory signaling network (Alers et al., 2012). The results showed that overexpression of APP695swe/TARDBP decreased p-AMPK activation (Figure 5a) and increased p-mTOR activation (Figure 5b). Figure 5c shows the results of the semi-quantitative analysis of Figures 5a,d, shows the results of the semi-quantitative analysis of Figure 5b. However, ICT treatment partially restored protein expression levels, suggesting that ICT can, to some extent, reverse the autophagy dysregulation induced by APP695swe/TARDBP through the AMPK/mTOR pathway. Furthermore, we performed molecular docking between TDP-43 protein and ICT to explore whether ICT could bind to TDP-43. The results indicated that ICT binds to TDP-43 with a binding energy of −7.1 kcal/mol, demonstrating a strong binding interaction between the two (Figure 6). Therefore, exploring the direct interaction between ICT and TDP-43 provides a direction for our future research.

## 4 Discussion

In the past decade, considerable efforts have been made to find new therapeutic approaches for AD with the aim of preventing or treating the disease. TDP-43 pathology is commonly observed in the brains of AD patients and is closely related to disease progression. It has been shown to affect AD pathology and shares a common genetic risk factor with AD, namely the apolipoprotein E4 (ApoE4) allele (Meneses et al., 2021). The mere presence of TDP-43 pathology increases the likelihood of developing AD (Meneses et al., 2021; Zhou et al., 2025; He et al., 2025; Huang W. et al., 2020), suggesting that TDP-43 pathology is an integral part of AD pathology. Therefore, targeting TDP-43 pathology holds promise as an effective therapeutic strategy for AD.

ICT, a flavonoid natural compound derived from the hydrolysis of icariin, is a bioactive compound with antioxidant, anti-neuroinflammatory, neuroprotective, and anti-aging properties (Wang et al., 2007; Jin et al., 2019; Zhang et al., 2021) due to its polyphenolic hydroxyl chemical structure (Zhang and Zhang, 2017). Previous studies have shown that ICT can alleviate cellular damage induced by Aβ (Li et al., 2024), Tau (Li et al., 2022a), and TDP-43 pathologies (Zhou et al., 2021; Zhou et al., 2022). However, whether ICT exerts therapeutic effects on AD models that combine TDP-43 pathology remains unknown. In this context, ICT may have an impact on TDP-43 pathology in AD. Our results confirm that the overexpression of TDP-43, leading to the formation of p-TDP-43 pathology, reduces cell viability, exacerbates Aβ42 secretion, and promotes Tau pathology formation. These results are consistent with previous reports, indicating that TDP-43 pathology can exacerbate the core pathology of AD (Tomé et al., 2023; Davis et al., 2017). Notably, ICT treatment improved cell viability in TDP-43 cellular models, attenuated p-TDP-43 aggregation, reduced Aβ42 levels, and alleviated Tau pathology, suggesting that ICT may exert broad protective effects against multiple AD-related pathologies.

Mitochondria play a central role in cellular biology and are essential for life, primarily responsible for energy production, calcium handling, apoptosis, and cell signaling (Klemmensen et al., 2024). Previous studies have shown that mitochondria are one of the primary targets of TDP-43 pathological proteins (Wang W. et al., 2016; Wang et al., 2019; Bennett et al., 2018). Consistent with prior reports (Wang W. et al., 2016; Wang et al., 2019), we observed that TDP-43 pathology led to mitochondrial swelling, significant loss of cristae, increased ROS generation, and decreased ATP production. ICT treatment partially reversed these damages: after 24 h of ICT exposure, mitochondrial morphology in TDP-43 cells was improved, cristae structure was enhanced, ROS levels were reduced, and ATP production increased. This mitochondrial protective effect aligns with our previous findings that ICT ameliorates TDP-43–induced mitochondrial dysfunction (Zhou et al., 2021), suggesting that maintaining mitochondrial integrity may be an important component of ICT’s neuroprotective mechanism. Notably, as shown by the results, ICT effectively reduced intracellular ROS levels; however, this effect does not appear to be strictly dose-dependent. Specifically, moderate doses of ICT significantly decreased ROS levels, whereas further increasing the dose did not enhance this effect and instead led to a partial rebound in ROS. This non-linear response suggests that the antioxidative effect of ICT may have an optimal dose range rather than increasing proportionally with dose.

Mitochondrial autophagy homeostasis is essential for maintaining normal mitochondrial function, cell survival, and overall cellular and biological functions (Pearson and Soleimanpour, 2018). The PINK1/Parkin pathway represents a classical ubiquitin-dependent mitophagy mechanism (Lu et al., 2023). When mitochondria are damaged, the mechanism by which PINK1 enters the inner mitochondrial membrane is blocked by the loss of mitochondrial membrane potential (MMP). Consequently, PINK1 binds to the outer mitochondrial membrane translocase and is retained on the outer mitochondrial membrane, while Parkin is recruited from the cytoplasm and undergoes a conformational change that activates it as an E3 ubiquitin ligase. This activation then leads to the aggregation of P62, which binds to the microtubule-associated protein light chain 3 (LC3), ultimately fusing with lysosomes to form an autolysosome (Riley et al., 2013). Previous studies have demonstrated that TDP-43 pathology can disrupt PINK1/Parkin-mediated mitophagy (Kato and Sakamoto, 2021). Consistent with these reports, our experimental results indicated that TDP-43 pathology activated the expression of PINK1/Parkin signaling pathway-related proteins. Additionally, ICT treatment was found to reduce the expression of these pathway-related proteins. The activation of the PINK1/Parkin pathway after TDP-43 overexpression may be associated with mitochondrial damage caused by TDP-43 pathology, which leads to excessive activation of mitochondrial autophagy. This suggests that ICT preserves mitochondrial function in neuronal cells by inhibiting the excessive activation of the PINK1/Parkin signaling pathway. Interestingly, our results showed that ICT treatment reduced PINK1 and Parkin levels in transfected cells, and in some cases even below the baseline observed in normal (non-transfected) cells. This phenomenon may reflect the restoration of mitochondrial homeostasis and alleviation of mitophagic stress rather than pathological suppression, as ICT mitigates cellular stress induced by the overexpression of disease-related proteins. Indeed, multiple studies have reported that moderate downregulation of PINK1 and Parkin does not impair mitochondrial function or induce detectable cellular toxicity. For example, no significant changes in brain mitophagy have been observed in PINK1 knockout *Drosophila* (Cummins and Götz, 2018; Han et al., 2020) or mice (Cummins and Götz, 2018; McWilliams et al., 2018), and loss of PINK1/Parkin signaling has not been shown to induce neurodegeneration in the brains of mice (Gautier et al., 2008; Kitada et al., 2009) or pigs (Zhou et al., 2015; Wang X. et al., 2016). These findings suggest that cells or organisms can tolerate a moderate reduction in PINK1/Parkin levels.

The mammalian nervous system, especially neurons, heavily relies on autophagy to clear large and insoluble protein aggregates, thereby maintaining protein homeostasis (Malampati et al., 2020). In our cell model, overexpression of TARDBP led to the formation of p-TDP-43 insoluble aggregates, which may disrupt the autophagic balance within the cells. As mentioned earlier, we observed that TDP-43 pathology further damages mitochondria and disrupts mitochondrial autophagy balance. Therefore, we aimed to further assess the impact of TDP-43 pathology on overall cellular autophagic activity.

The AMPK/mTOR pathway is a critical signaling pathway in autophagy. Autophagy driven by AMPK serves as a key sensor for regulating cell metabolism and maintaining energy homeostasis, with AMPK activation negatively regulating autophagy through the mTOR (Guo et al., 2021; Xia et al., 2024). Additionally, the AMPK/mTOR autophagy pathway plays an important role in AD (Salminen et al., 2011), and its regulation has been shown to improve AD-related pathology and cognitive deficits (Ou et al., 2018). Thus, ICT may alleviate cellular damage induced by TDP-43 pathology by modulating the AMPK/mTOR signaling pathway. In this study, we found that TDP-43 pathology inhibited AMPK activation while promoting mTOR activation, whereas ICT treatment restored the levels of these pathway proteins. This suggests that ICT improves autophagic function by modulating the AMPK/mTOR autophagy pathway, thereby reducing cellular stress and damage and alleviating TDP-43–mediated cytotoxicity. These findings are consistent with previous reports in other disease models showing that ICT can regulate the AMPK/mTOR signaling pathway (Zhao et al., 2020).

Overall, this study demonstrates that ICT exerts neuroprotective effects in cellular models harboring both AD and TDP-43 pathologies. The underlying mechanisms may involve the maintenance of mitochondrial function, suppression of excessive mitophagy via the PINK1/Parkin pathway, and regulation of overall autophagic responses through the AMPK/mTOR pathway. Molecular docking analysis further revealed a potential binding between ICT and TDP-43, providing a mechanistic basis for its potential direct action.

**Figure 7:**
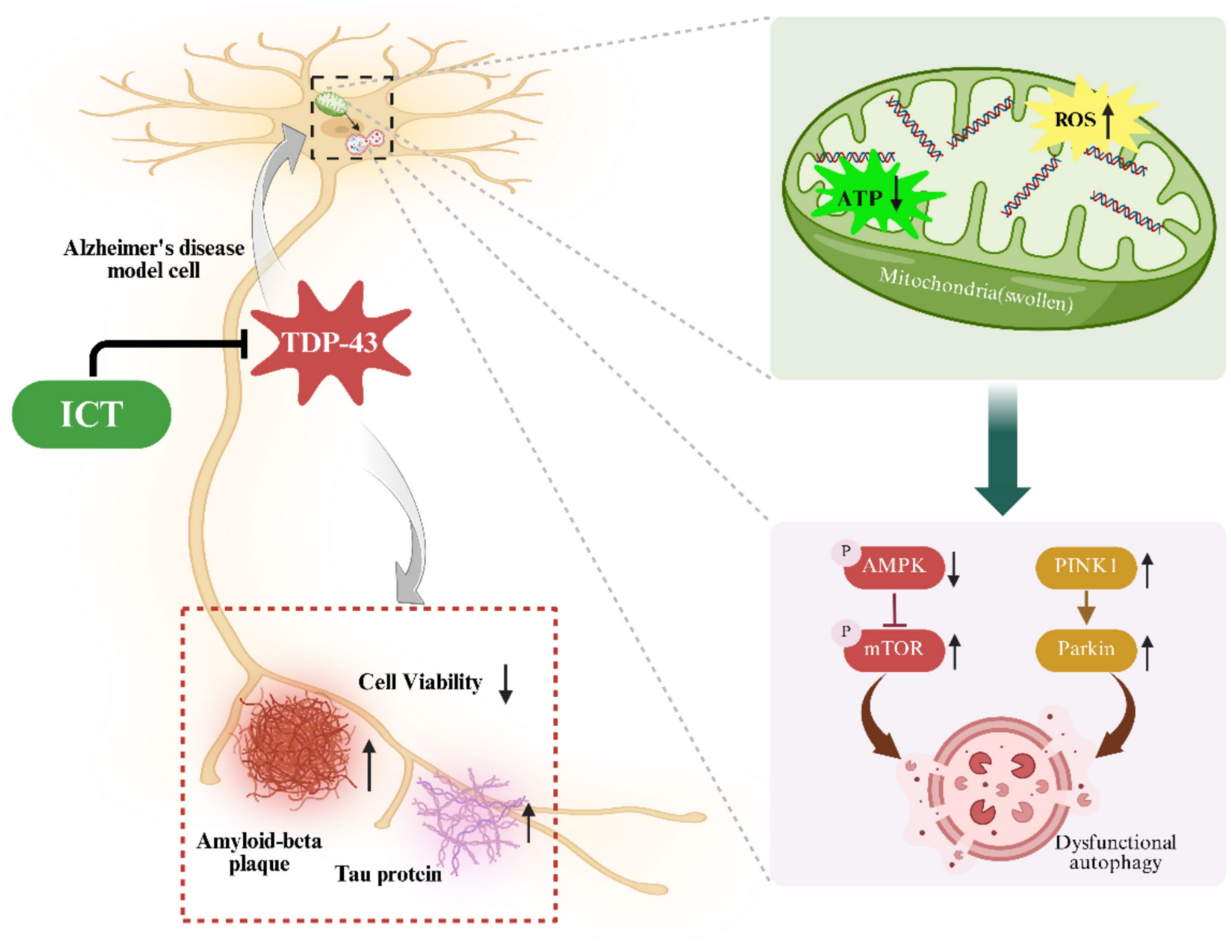
Schematic illustration of the protective effects of ICT against TDP-43 overexpression–induced mitochondrial damage and autophagy pathway dysfunction. Created with BioRender.com.

Despite these encouraging results, this study has several limitations: (1) we primarily focused on the AMPK/mTOR and PINK1/Parkin pathways but did not assess classical autophagy markers such as LC3 and p62; future studies will include these experiments. (2) TEM images were captured at high magnification and focused mainly on mitochondrial ultrastructure, so whole-cell images were not obtained, preventing accurate quantitative analysis of mitochondrial number. In addition, TEM images for the TDP-43 + 0.01 μM ICT group were not captured, which limits the completeness of the ultrastructural comparison across all treatment conditions. (3) Although *in vitro* cellular models provide mechanistic insights, the therapeutic potential of ICT still needs to be validated in animal models and clinical studies. (4) Considering the favorable pharmacological properties of ICT and the preliminary evidence of its neuroprotective effects, future research should further explore its translational potential in AD and related TDP-43 proteinopathies (see Figure 7).

## 5 Conclusion

ICT may exert a protective effect on mitochondrial damage and autophagy dysregulation induced by overexpression of TDP-43 in APP cells through the PINK1/Parkin and AMPK/mTOR signaling pathways.

## Data Availability

The raw data supporting the conclusions of this article will be made available by the authors, without undue reservation.
