# Supplementary figures and images for: Icaritin ameliorates mitochondrial dysfunction and autophagy impairment in cellular models of Alzheimer’s disease
Source: Front Aging Neurosci. 2026 Mar 10;18:1741339. doi: 10.3389/fnagi.2026.1741339 (PMC13008745; doi:10.3389/fnagi.2026.1741339)

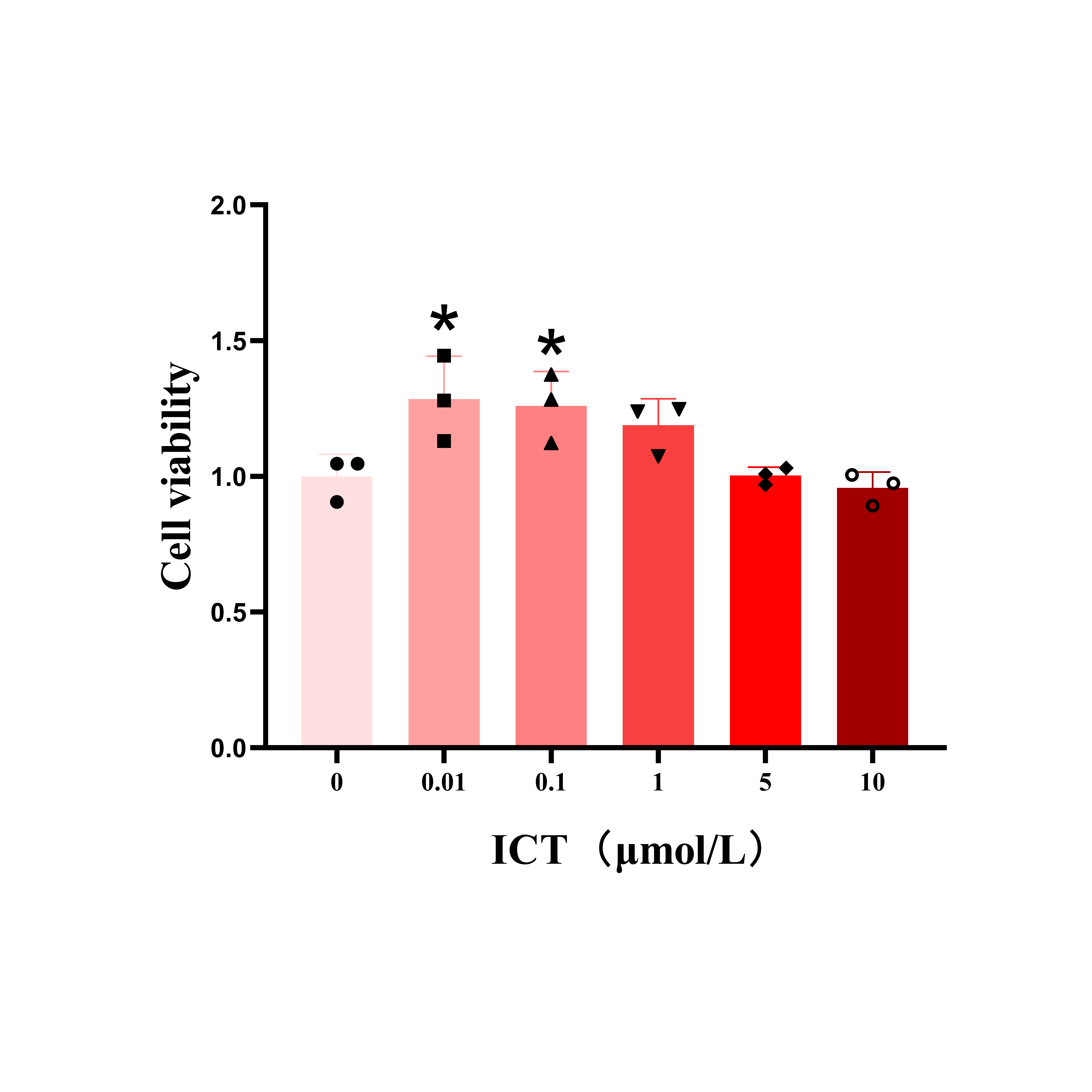

Supplement: Supplementary file 1 [file Supplementary_file_1.tif]
